# Ursodeoxycholic Acid May Inhibit Environmental Aging-Associated Hyperpigmentation

**DOI:** 10.3390/antiox10020267

**Published:** 2021-02-09

**Authors:** Ik Jun Moon, Hanju Yoo, Seung Hwan Paik, Hak Tae Kim, Su Yeon Kim, Youngsup Song, Sung Eun Chang

**Affiliations:** 1 Department of Dermatology, Asan Medical Center, University of Ulsan College of Medicine, Seoul 05505, Korea; ikjun.moon@gmail.com (I.J.M.); julia_yoo@hanmail.net (H.Y.); httkmd@gmail.com (H.T.K.); u2u2star@naver.com (S.Y.K.); 2 Bio-Medical Institute of Technology (BMIT), University of Ulsan College of Medicine, Seoul 05505, Korea; 3 Seoul ONE Dermatology Clinic, Seoul 05505, Korea; 2kilo1mine@hanmail.net; 4 Department of Biomedical Sciences, Asan Medical Center, University of Ulsan College of Medicine, Seoul 05505, Korea

**Keywords:** ursodeoxycholic acid, antioxidant, photoaging, environmental aging, particulate matter, hyperpigmentation, fibroblasts, ultraviolet light

## Abstract

Extrinsic aging of the skin caused by ultraviolet (UV) light or particulate matter is often manifested by hyperpigmentation due to increased melanogenesis in senescent skin. Ursodeoxycholic acid (UDCA), which has been commonly used as a health remedy for liver diseases, is known to possess antioxidant properties. This study was done to investigate whether UDCA inhibits cellular aging processes in the cells constituting human skin and it reduces melanin synthesis. ROS, intracellular signals, IL-1α, IL-8, TNF-α, cyclooxygenase (COX)-2, type I collagen, and matrix metalloproteinases (MMPs) levels were measured in human dermal fibroblasts treated with or without UDCA after UV exposure. Melanin levels and mechanistic pathways for melanogenesis were investigated. UDCA decreased ROS, senescence-associated secretory phenotype (SASP), and proinflammatory cytokines induced by UV treatment. UDCA reduced melanogenesis in normal human melanocytes cocultured with skin constituent cells. Our results suggest that UDCA could be a comprehensive agent for the treatment of environmental aging-associated hyperpigmentation disorders.

## 1. Introduction

Skin aging processes can be divided into intrinsic and extrinsic aging processes [1,2]. Intrinsic aging refers to aging caused only by internal factors, also called chronologic aging. In contrast, extrinsic aging refers to aging caused by external factors, including ultraviolet (UV) radiation, smoke, and airborne pollutants such as particulate matter (PM). Accelerated skin aging due to these exogenous factors involve a common molecular event known as increased oxidative stress [3]. Among other organs, the skin is highly vulnerable to aging caused by extrinsic factors because it is constantly in direct contact with the environment.

UV-induced aging, also referred to as photoaging, is the main focus of aging studies, because the negative effects of UV radiation on the skin have been extensively documented at the molecular level, making the prevention of photoaging possible. In photoaged skin, a profoundly decreased amount of dermal collagen is observed, causing more severe wrinkles compared with intrinsic aging. UV-induced cellular senescence of the constituent cells of the skin, including dermal fibroblasts, has a role in the mechanism of skin photoaging [4,5]. In Asian skin, uneven pigmentation and skin tone darkening are particularly troublesome aspects of photoaging, since UV radiation increases the synthesis of melanin, causing common hyperpigmentation disorders such as melasma, post-inflammatory hyperpigmentation, and solar lentigines [6]. In Asians, the first signs of photoaging are photoaging-associated mottled pigmentation (mottling) and solar lentigines occurring as early as 20 years of age [7].

Extrinsic aging can also be attributed to multiple factors other than UV radiation, such as exposure to PM. As the aggravation of air pollution has become a global issue, the detrimental effects of exposure to airborne pollutants have been actively investigated, and recent reports have suggested that exposure to PM may result in cutaneous hyperpigmentation as well as skin aging [8,9]. Although the exact molecular process implicated in PM-induced skin aging and hyperpigmentation, an increase in oxidative stress, which is also important in UV-induced aging, has been suggested to be a key process [10].

Research on the development of skin lightening agents is still a priority of dermatological, cosmeceutical, and nutraceutical investigators in darker-skinned races [11]. Nevertheless, mere depigmenting agents targeting epidermal melanocytic tyrosinase activity are far from satisfactory, because extrinsic aging-associated hyperpigmentation should be addressed to reverse the extrinsic aging of the dermal stroma. Senescent human fibroblasts induce melanogenesis in skin equivalents [12], and dermal fibroblasts have an active role in the skin pigmentation system by secreting several paracrine factors to activate epidermal melanocytes [13].

Oral administration of ursodeoxycholic acid (UDCA), a secondary bile acid, has been commonly used for the prevention and treatment of cholestatic or toxic liver diseases. UDCA shows anti-oxidant, anti-apoptotic, and anti-inflammatory properties, and it has been considered safe for several decades [14,15]. The use of UDCA is extending to non-cholestatic and non-hepatic diseases because of its multiple beneficial health-promoting mechanisms [16,17,18]. Thus, we thought that UDCA might ameliorate extrinsic aging-associated hyperpigmentation. Therefore, we investigated whether UDCA inhibits cellular aging in fibroblasts and reduces melanin synthesis in a coculture of human melanocytes with simulated human skin constituent cells.

## 2. Materials and Methods

### 2.1. Cell Culture

Normal human epidermal melanocytes (NHMs) (Invitrogen, Carlsbad, CA, USA) at passage 3–5 were cultured in medium 254 supplemented with human melanocyte growth supplement (Invitrogen, Carlsbad, CA, USA). B16F10 murine melanoma cells were maintained in DMEM (Gibco-BRL, Bethesda, MD, USA) containing 10% fetal bovine serum. Human dermal fibroblasts (HDFs) from adult skin were cultured at passages 2–3 in medium 106 supplemented with low serum growth supplement (Invitrogen, Carlsbad, CA, USA). In the coculture of NHMs and HDFs, NHMs (1.5 × 10^5^) were seeded in the inserts of Transwell chambers (Corning, Tewksbury, MA, USA), and HDFs (3 × 10^5^) were seeded at the bottom of the 6-well plates. After 24 h (h) of starvation, HDFs were irradiated with UVB 20 mJ/cm^2^. Then, the insert chambers were moved into the HDF-seeded 6-well plates, and the cultures were maintained in fibroblast culture medium for 3 days to measure the melanin content. Coculture of NHMs and normal human keratinocytes (NHKs) was generated in keratinocyte medium, at a seeding ratio of 1:5 (for melanin assays) or 1:1 (for melanin assays, Western blotting and intracellular signaling assays). NHMs were seeded into a 6-well plate at a density of 6 × 10^4^ or 3 × 10^5^ cells per well. On the next day, NHKs were added to each well at a density of 3 × 10^5^ cells for the coculture. Ursodeoxycholic acid (UDCA), dissolved in ethanol, was provided by Daewoong Pharmaceutical Company (Seoul, Korea).

### 2.2. Measurement of Melanin Content

Melanin contents were measured using the method previously described by Moon, with a slight modification [19]. In brief, B16F10 cell and NHMs were cultured in 6-well plates at a density of 1 × 10^5^ and 6 × 10^5^, respectively. Then the cells were dissolved in 1 N NaOH at 100 °C for 30 min (min) and centrifuged at 13,000 rpm for 5 min. The optical densities of the supernatants were measured at an absorbance of 405 nm using a microplate reader. The amount of protein in the sample was measured using the Bradford assay (Bio-Rad, Hercules, CA, USA). Melanin content was normalized to the protein amount. Kojic acid and β-arbutin (4-hydroxyphenyl-β-D-glucopyranoside) were purchased from Sigma Aldrich Co. (St. Louis, MI, USA), and used as positive controls at concentrations of 100 μM and 50 μM, respectively. All measurements of the melanin content were performed on the third day of incubation.

### 2.3. Intracellular Tyrosinase Activity Assay

The intracellular tyrosinase activity assay was performed using the method described by Moon [19]. NHMs were cultured in 6-well plates at a density of 6 × 10^5^. The cells were treated with 50 and 100 μM UDCA for 5 days, and then the cells were lysed in phosphate buffer (pH 6.8) containing 1% Triton X-100. The protein levels of the lysate were measured. Following adjustment of the protein concentrations with lysis buffer, the lysate was treated with 5 mM L-DOPA. After incubation at 37 °C, tyrosinase activity was measured with a microplate reader at 475 nm.

### 2.4. Exposure to UV Radiation, Particulate Matter (PM), or Growth Factors

The cells were exposed to UVA with a PL-S 9W lamp (Philips, Eindhoven, The Netherlands) and a Dermalight (National Biological Corp., Twinsburg, OH, USA) or UVB with a TL20W/12RS UV lamp (Philips, Eindhoven, The Netherlands). Similar to the previously published research on UVB exposure, the cells were starved for 24 h and washed in phosphate-buffered saline (PBS) before exposure to UVA and UVB radiation [20,21,22,23]. Non-exposed control samples were maintained in the dark under the same conditions. Following exposure to UVA or UVB radiation, the cells were grown in culture medium and treated with UDCA. For the UVA irradiation, a UV Crosslinker (Ultra-Violet Products Ltd., Cambridge, UK) was used, with a UV spectrum of 365 nm (UV-A) and 302 nm (UV-B). After 24 h, the medium was removed, the cells were washed with PBS twice, and then the PBS was removed. We collected and selected local PM with a particle size less than 10 μm (by reference to the previous publication of Jin et al. [24]) outside the Asan Research Institution building located in an urban area in Seoul, Korea from January 2019 to March 2019. The collection site was 200 m away from a two-way street with total of eight lanes. Han River, which is more than one kilometer wide, is located 500 m away from the collection site. For treatment with local PM, cells were treated with 100 μg/mL PM for 24 h, then the medium was removed and cells were washed twice with PBS. The cells were stimulated with stem cell factor (SCF) (R&D Systems, Minneapolis, MN, USA) or endothelin-1 (ET-1) (Sigma Aldrich Co., St. Louis, MI, USA) for 3 days.

### 2.5. 2′,7′-Dichlorofluorescein Diacetate (DCF-DA) Microplate Assay

Intracellular reactive oxygen species (ROS) levels were measured by the DCF-DA (CELL BIOLABS, Inc., San Diego, CA, USA) assay, according to the manufacturer’s instructions. Briefly, HDFs were seeded in a 96-well plate at a rate of 5 × 10^3^ cells per well and treated with 10 or 50 μM UDCA or 50 μM vitamin C (Sigma-Aldrich Co., St. Louis, MI, USA) for 24 h. NHKs were treated with different combinations of 100 μg/mL PM, 100 μM UDCA, and 0.5 or 1 mM N-acetylcysteine (Sigma Aldrich Co., St. Louis, MI, USA) for 24 h. Cells were incubated with 10 μM DCF-DA for 30 min at 37 °C in the dark and washed with PBS twice. Then, the fluorescence was detected at 480 nm excitation and 530 nm emission using a spectrofluorometer (SpectraMax i3, Molecular Devices, Sunnyvale, CA, USA).

### 2.6. Quantitative Real-Time Polymerase Chain Reaction (qRT-PCR)

Total cellular RNA was extracted from the cells using a FavorPrepTM Total RNA Purification Mini Kit according to the manufacturer’s instructions (Favorgen, Ping-Tung, Taiwan). Following isolation, the quantity and quality of the RNA were determined using a NanoDrop^®^ ND-1000 Spectrophotometer (ND-1000, NanoDrop Technologies, Wilmington, DE, USA). Single-stranded cDNA was synthesized from 1 μg of total RNA using a Revert Aid First Strand cDNA Synthesis Kit according to the manufacturer’s instructions (Thermo Scientific, Rockford, IL, USA). qRT-PCR was performed using a LightCycler^®^ 480II machine coupled with SYBR Green chemistry (Roche Applied Science, Penzberg, Germany). In terms of qRT-PCR settings, initial denaturation was performed at 95 °C for 5 min, followed by amplification at 95 °C for 10 s, 60 °C for 10 s, and 72 °C for 10 s for 45 cycles. The cDNA obtained was amplified with the primers listed in Table 1.

### 2.7. Western Blotting

Cells were lysed in protein lysis buffer (Intron Biotechnology, Seongnam, Korea) and centrifuged at 13,000 rpm for 10 min. Protein concentrations were determined using a bicinchoninic acid protein assay kit. Next, 20 μg of protein per lane was separated by SDS-polyacrylamide gel electrophoresis and blotted onto nitrocellulose membranes. Blots were incubated with the appropriate primary antibodies at a dilution of 1:1000 and then further incubated with horseradish peroxidase-conjugated secondary antibodies. Bound antibodies were detected using an enhanced chemiluminescence kit (Pierce Biotechnology, Rockford, IL, USA). Image analysis was performed using Image J software (https://imagej.nih.gov/ij/ accessed on 28 December 2020) to determine the relative band densities. Antibodies specific for type I collagen and tyrosinase were purchased from Santa Cruz Biotechnology, Inc. (Santa Cruz, CA, USA), and antibodies specific for total extracellular signal-regulated kinase (ERK), phospho-ERK, total p38, phospho-p38, total c-Jun N-terminal kinase (JNK), phospho-JNK, and phospho-c-Jun were purchased from Cell Signaling Technology (Danvers, MA, USA). Antibodies specific for cyclooxygenase-2 (COX-2) were purchased from Abcam (Cambridge, UK). Tyrosinase and microphthalmia-associated transcription factor (MITF) were purchased from Thermo Fisher Scientific (Cheshire, UK), and actin was purchased from Sigma-Aldrich Co. (St. Louis, MO, USA).

### 2.8. MTT Assay

Cell viability was measured using MTT assays. All cells were treated with 10–200 µM of UDCA for 3 days. MTT solution (2.5 µg/mL) was added to the culture medium and incubated for 4 h. MTT staining was extracted with DMSO. Absorbance was determined using a microplate reader (Molecular Devices, Sunnyvale, CA, USA) at 570 nm.

### 2.9. Statistical Analysis

The statistical significance of the differences between groups was assessed by ANOVA, followed by Student’s t-test. *P* < 0.05 and *P* < 0.01 were considered statistically significant.

## 3. Results

### 3.1. Antioxidant Property of UDCA

#### 3.1.1. UDCA Decreases ROS Levels Induced by UVA and UVB in HDFs

**Figure 1:**
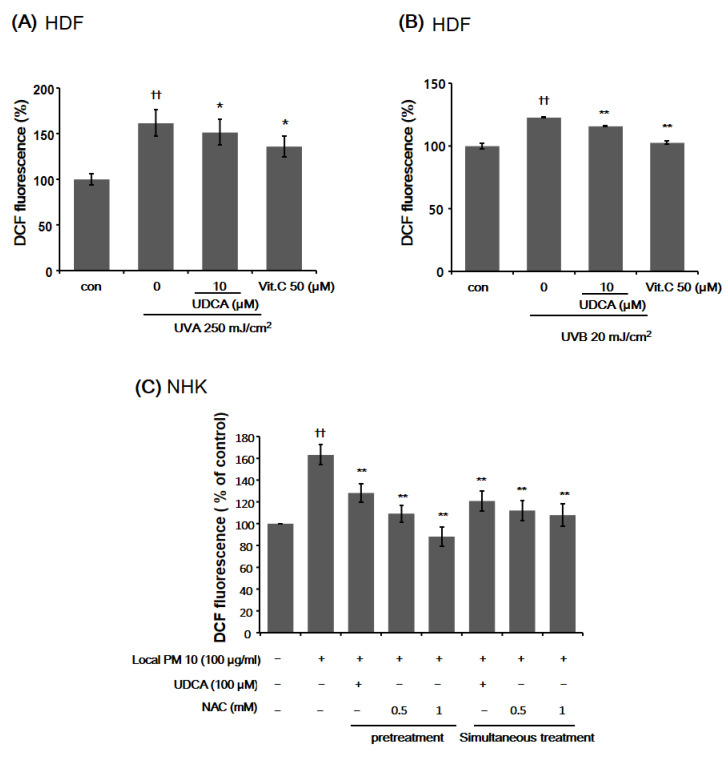
The effect of ursodeoxycholic acid (UDCA) on intracellular oxidative stress. (**A**) Exposure of human dermal fibroblasts (HDFs) to 250 mJ/cm^2^ of UVA resulted in a significant increase in intracellular oxidative stress, which was attenuated by treatment with 10 μM UDCA. (**B**) Similarly, exposure of HDFs to 20 mJ/cm^2^ of UVB resulted in a significant increase in intracellular oxidative stress, which was effectively mitigated by treatment with 10 μM UDCA. Treatment with 50 μM of vitamin C was used as a positive control. (**C**) The effect of UDCA treatment on the intracellular oxidative stress of normal human keratinocytes following exposure to particular matter (PM). Three hours after treatment with 100 μg/mL of local PM10, NHKs were either pretreated or treated simultaneously with UDCA. Both modes of UDCA treatment resulted in a significant reduction of oxidative stress, measured by DCF fluorescence. Treatment with N-acetylcysteine (NAC) was used as a positive control. * *p* < 0.05 and ** *p* < 0.01 compared with UV-exposed or PM-treated control, ^††^ *p* < 0.01 compared to control (con).

Low doses of both UVA and UVB increased the ROS levels in HDFs (Figure 1A,B). DCF fluorescence in human dermal fibroblasts (HDFs) treated with vitamin C as a positive control was decreased compared to that of the untreated control. Increased DCF fluorescence after UVA exposure decreased in the HDFs treated with 10 µM UDCA compared to the untreated control (Figure 1A). Increased DCF fluorescence after UVB exposure also decreased in the HDFs treated with 10 µM UDCA compared to the untreated control (Figure 1B).

#### 3.1.2. UDCA Attenuates the Increased ROS Level Following Exposure to PM in NHKs

In the DCF assay using normal human keratinocytes (NHKs), a notable increase in ROS level was observed 24 h after exposure to local PM (Figure 1C). However, this increase in intracellular oxidative stress was effectively attenuated by both pretreatment and simultaneous treatment with 100 µM UDCA. The degree of ROS downregulation did not differ significantly between pretreatment and simultaneous treatment.

### 3.2. Anti-Inflammatory Property of UDCA

#### 3.2.1. UDCA Treatment Had an Anti-Inflammatory Effect against the Inflammatory Cellular Microenvironment Resulting from Exposure to UV or PM

**Figure 2:**
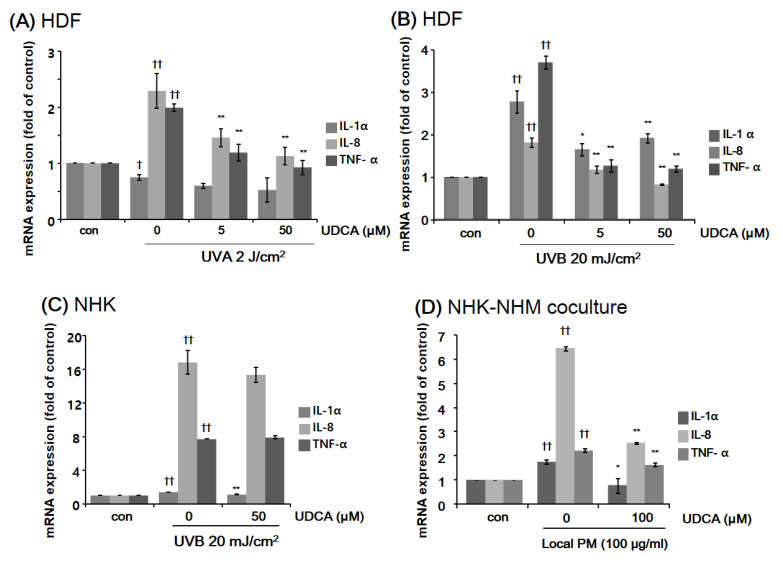
Anti-inflammatory effect of UDCA treatment. HDFs were exposed to (**A**) UVA and (**B**) UVB, which both induced incremental changes in mRNA expression of proinflammatory cytokines, including IL-1a, IL-8, and TNF-α. Downregulation of inflammatory cytokines was more prominent when HDFs were treated with 50 μM UDCA as compared with 10 μM UDCA. (**C**) Proinflammatory cytokine expression of normal human keratinocytes (NHKs) was also increased following exposure to UVB. However, IL-8 and TNF-α were not decreased by treatment with 50 μM UDCA. (**D**) In a coculture consisting of NHKs and normal human melanocytes (NHMs), treatment with 100 μM UDCA effectively downregulated the elevated mRNA expression levels of proinflammatory cytokines following exposure to local PM10. * *p* < 0.05 and ** *p* < 0.01 compared with UV-exposed or PM-treated control. ^†^ *p* < 0.05 and ^††^ *p* < 0.01 compared with the null control (con).

The inhibitory effect of UDCA against inflammatory cytokines in HDFs was determined using RT-PCR. After treatment with UDCA (5, 50 μM) and exposure to 2 J/cm^2^ of UVA for 6 h, proinflammatory cytokine levels were measured. UDCA reduced the amounts of interleukin (IL)-8 and tumor necrosis factor-α (TNF-α) increased by UVA (Figure 2A). Proinflammatory cytokine levels of HDFs exposed to UVB 20 mJ/cm^2^ and treated with UDCA for 3 h were measured. UDCA reduced IL-1α, IL-8, and TNF-α RNA expression increased significantly in response to UVB (Figure 2B). Then the anti-inflammatory effect of UDCA was tested on NHKs. As shown in Figure 2C, treatment with 50 µM UDCA reduced the level of IL-1α, which was upregulated following the exposure to low-dose UVB. However, UVB-induced elevations in IL-8 and TNF-α levels did not drop significantly after treatment with UDCA. Next, the effect of UDCA treatment on the expression of proinflammatory cytokines in a coculture of NHKs and NHMs following exposure to local PM was assessed. As shown in Figure 2D, the expression of all three tested proinflammatory cytokines were elevated after exposure to PM. Treatment with 100 µM UDCA resulted in successful mitigation of this increase in proinflammatory cytokine expression.

#### 3.2.2. UDCA Reduces the Expression of Proteins Associated with Environmental Aging and Inflammation in HDFs While Restoring Type I Collagen Expression

**Figure 3:**
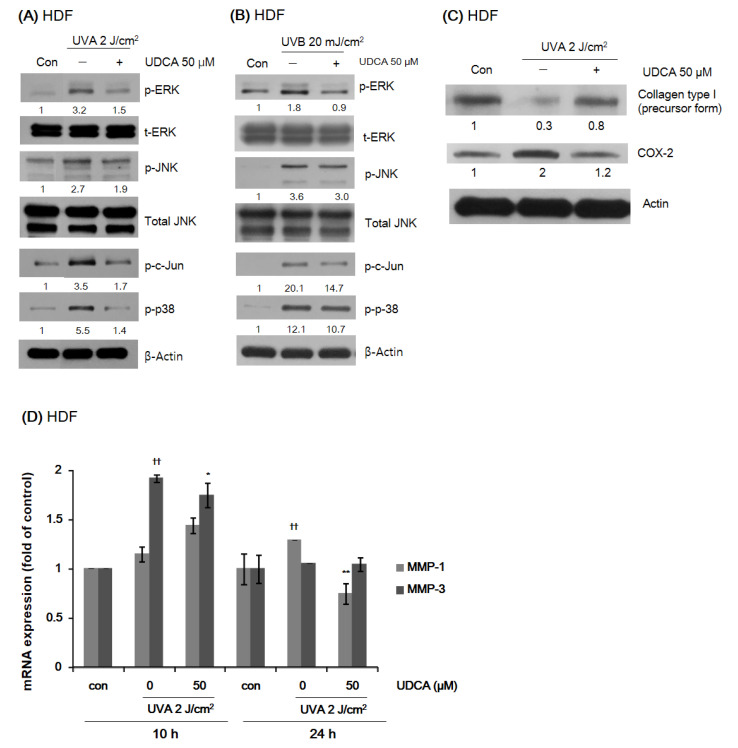
Effect of UDCA treatment on the expression of phosphorylated proteins related to environmental aging-associated inflammation following exposure to (**A**) UVA and (**B**) UVB in HDFs. For both spectrums of UV radiation, treatment with 50 μM UDCA resulted in decreased expression of p-ERK, p-JNK, p-c-Jun, and p-p-38. (**C**) Changes in expression levels of type 1 collagen and COX-2, as a senescence-associated secretory phenotype, after exposure to UVA, and the effect of UDCA treatment. Treatment with 50 μM UDCA increased the expression of type I collagen, while it decreased the expression of COX-2. (**D**) Inhibitory effect of UDCA on MMP-1 and MMP-3 expression induced by UVA in HDFs. * *p* < 0.05 and ** *p* < 0.01 compared with the UV-exposed control. ^††^ *p* < 0.01 compared with the null control (con).

The expression levels of phosphorylated proteins associated with environmental aging and inflammation in HDFs were measured by Western blot analysis after treatment with 50 µM UDCA for 1 h and exposure to 2 J/cm^2^ UVA and 20 mJ/cm^2^ UVB for 1 h. UDCA treatment reduced the expression of phospho-ERK, phospho-JNK, phospho-c-Jun, and phospho-p38, which were increased by both UVA (Figure 3A) and UVB (Figure 3B) irradiation. The densitometric values were normalized to the expression of their total forms or β-actin. To examine the effect of UDCA on type I collagen expression and COX-2 altered by UVA, HDFs were treated with UDCA for 24 h after UVA irradiation. UDCA increased the expression of type I collagen reduced by UVA irradiation and decreased COX-2 induced by UVA irradiation (Figure 3C). We further tested the effect of UDCA on the expression of MMP-1 and MMP-3 that were increased by UVA irradiation using RT-PCR. The expression levels of MMP-1 and MMP-3 were evaluated 10 h and 24 h after UVA irradiation, which were their peak expression times. UDCA treatment reduced the increase in MMP-1 and MMP-3 expression induced by UVA (Figure 3D).

### 3.3. Anti-Melanogenic Property of UDCA

#### 3.3.1. UDCA Decreases Melanogenesis in Human Melanocytes

**Figure 4:**
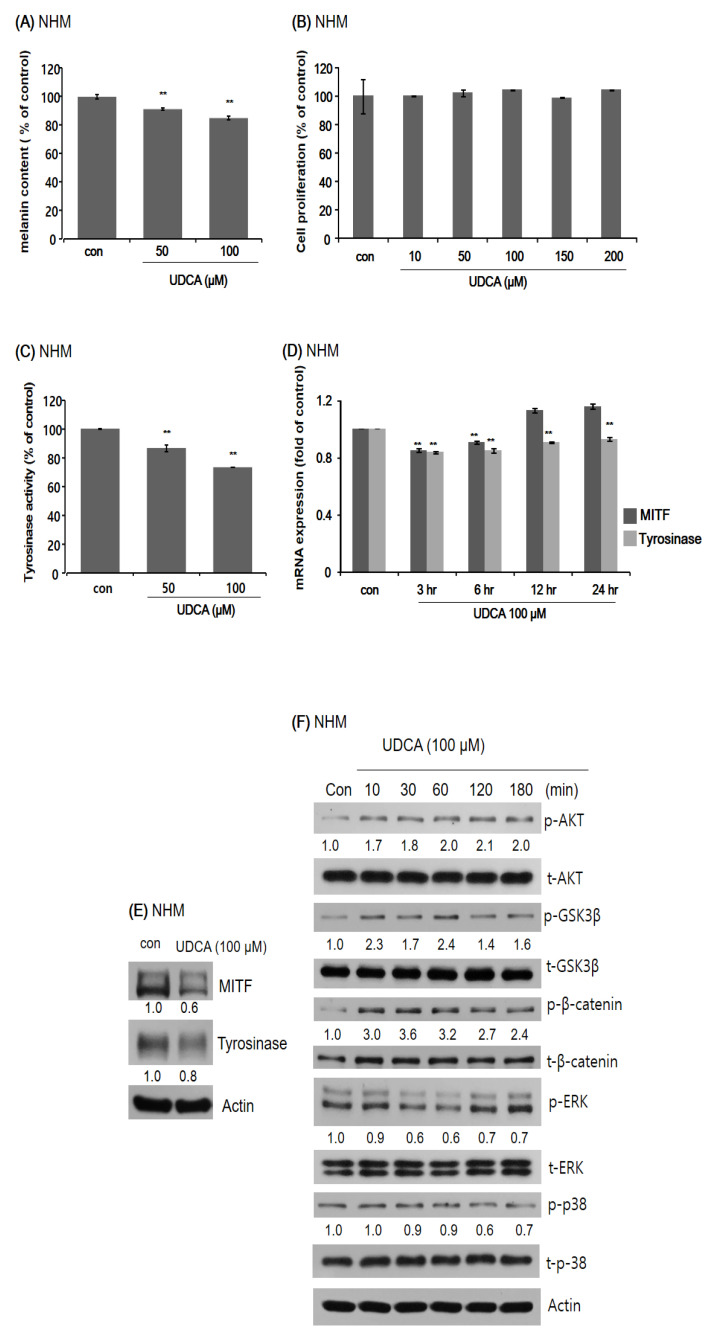
The effect of UDCA on normal human melanocytes (NHMs). (**A**) Treatment with 50 and 100 μM UDCA resulted in decreased melanin content in NHMs. (**B**) NHMs were treated with different concentrations of UDCA from 10 μM to 200 μM. Cell proliferation was not affected by the tested concentrations of UDCA. (**C**) Treatment with 50 and 100 μM resulted in decreased tyrosinase activity in NHMs. (**D**) Expression of MITF and tyrosinase in NHMs. Treatment with 100 μM UDCA effectively lowered the mRNA expression levels of both MITF and tyrosinase starting from 3 h after treatment. By 12 h after treatment, recovery of mRNA expression of MITF was observed whereas that of tyrosinase suppressed until 24 h after treatment. (**E**) The results of Western blotting show decreased levels of both MITF and tyrosinase following UDCA treatment at 24 h. (**F**) Effect of UDCA treatment on the expression of melanogenesis-related signaling proteins in NHMs. Treatment with 100 μM UDCA resulted in increased levels of p-AKT, p-GSK3β, and p-β-catenin, and decreased levels of p-ERK and p-p-38. ** *p* < 0.01 compared with the non-UDCA-treated control.

As the first step to determining the anti-melanogenic effects of UDCA, we observed a significant decrease in melanin content when normal human melanocytes (NHMs) were treated with UDCA (Figure 4A). Next, an MTT assay was carried out to demonstrate that the cell viability of NHMs was not affected by UDCA treatment (Figure 4B). UDCA reduced the melanin content in a dose-dependent manner (Figure 4B). Knowing that UDCA is not toxic to NHMs, we examined the changes in tyrosinase activity induced by UDCA treatment. As shown in Figure 4C, treatment with 50 and 100 µM both led to a decreased tyrosinase activity. Then the effect of UDCA treatment on the mRNA expression as well as protein levels of MITF and tyrosinase were tested using qRT-PCR and Western blot analysis, respectively. As demonstrated in Figure 4D,E, both mRNA expression and protein levels of MITF and tyrosinase were downregulated by 100 µM UDCA. Finally, changes in the expression of melanogenesis-related signaling proteins in NHMs induced by UDCA treatment were assessed by Western blotting. Treatment with UDCA resulted in increased levels of p-AKT, p-GSK3β, and p-β-catenin, and decreased levels of p-ERK and p-p-38 (Figure 4F).

#### 3.3.2. UDCA Decreases the Melanin Content in B16F10 Cells and HDF-NHM Coculture under Stimulated Conditions

**Figure 5:**
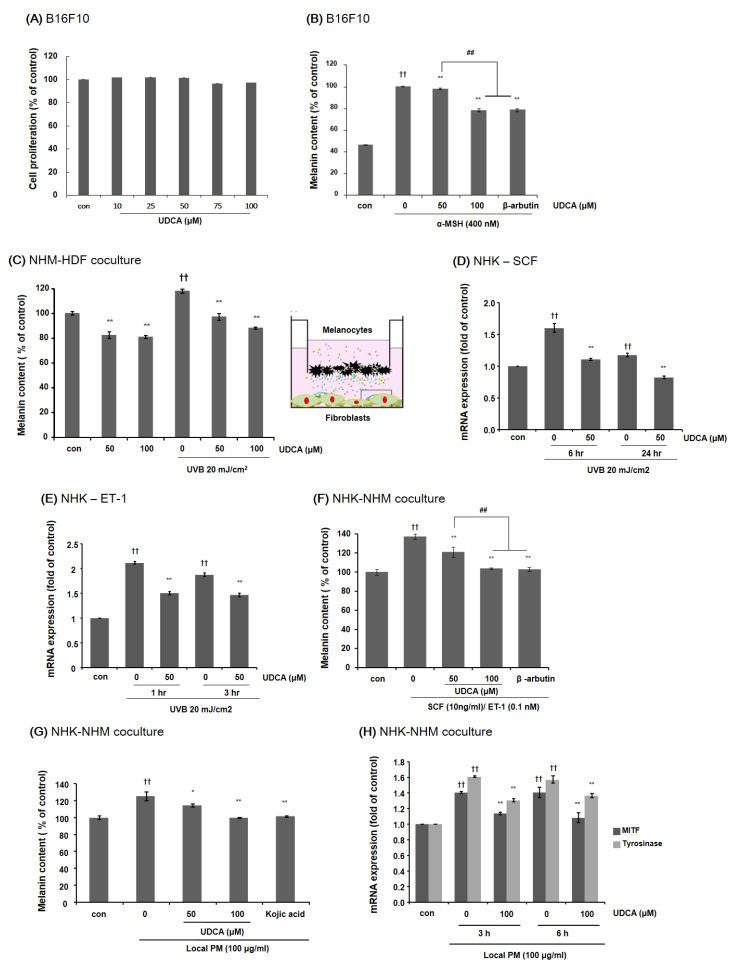
The effect of UDCA treatment on B16F10 cells and coculture. (**A**) B16F10 cells were. treated with different concentrations of UDCA. No change in cell proliferation was observed. (**B**) Treatment of B16F10 cells with UDCA could effectively reduce the melanin content, which was induced by α-MSH. Arbutin was used as a positive control. (**C**) Treatment with UDCA resulted in a significant decrease in melanin content both in the presence and absence of UVB exposure in NHM-HDF coculture. Treatment with 50 μM UDCA downregulated the mRNA expression of both (**D**) SCF and (**E**) ET-1 in NHKs following exposure to UVB. (**F**) Treatment with UDCA resulted in a significant decrease in melanin content both in the presence and absence of UVB exposure in NHM-HDF coculture. (**G**) Exposure of HNK-NHM coculture to local PM significantly increased the melanin content. However, treatment with UDCA effectively reduced the melanin content. Kojic acid was used as a positive control. (**H**) Exposure of HNK-NHM coculture to local PM led to an increased expression of MITF and tyrosinase, which was significantly attenuated by treatment with 100 μM of UDCA. * *p* < 0.05, ** *p* < 0.01 compared with the counterpart not treated with UDCA, ^††^ *p* < 0.01 compared with the control (con), ^##^ *p* < 0.01.

First, MTT assays were performed to demonstrate that UDCA treatment does not impact B16F10 cell viability. As shown in Figure 5A, treatment with 10 to 100 µM UDCA did not affect B16F10 cell proliferation. Then, in order to measure the effects of UDCA on extrinsic aging-associated hyperpigmentation, B16F10 melanoma cells were treated with UDCA in the presence of melanogenic stimulation by α-MSH. As demonstrated in Figure 5B, UDCA could effectively reduce the melanin content induced by α-MSH. The NHMs were cocultured with HDFs that were exposed to UVB 20 mJ/cm^2^. The melanin content of the UV-exposed coculture was higher compared with the non-irradiated control. UDCA successfully reduced the melanin content in the coculture regardless of UV exposure (Figure 5C). Then we checked for the effect of UDCA on the mRNA expression of stimulatory molecules of melanin synthesis. As we have already established that UVB treatment of NHK-NHM coculture stimulates the production of SCF and ET-1 based on our previous studies, we have tested whether UDCA treatment can downregulate their expression [19]. As expected, treatment with UDCA at a concentration of 50 µM decreased the mRNA levels of both SCF and ET-1 in UVB-irradiated NHKs (Figure 5D,E, respectively). NHMs were cocultured with NHKs under stimulation with 10 ng/mL of SCM and 0.1 nM of ET-1. As shown in Figure 5F, treatment with both 50 and 100 µM UDCA could significantly lower the melanin content of the coculture. In particular, treatment with 100 µM UDCA reduced the melanin content to a degree equivalent to treatment with a well-known inhibitor of melanogenesis, arbutin.

#### 3.3.3. UDCA Attenuates the Increase in Melanin Content Induced by Treatment with PM in NHK-NHM Coculture

Finally, we investigated whether UDCA could mitigate the increase in melanin content stimulated by exposure to PM. A significant increase in melanin content was observed in NHK-NHM coculture when exposed to 100 µg/mL of local PM. However, treatment with UDCA could effectively reduce this increase in melanin content (Figure 5G). Treatment with local PM resulted in a notable increase in expression levels of both MITF and tyrosinase, but simultaneous treatment with UDCA significantly reduced the mRNA expression levels of both genes (Figure 5H).

## 4. Discussion

Extrinsic aging is characterized by the degradation of collagen fibers, which causes skin wrinkling and increased melanocytic activity leading to hyperpigmentation [4,5]. A number of environmental factors have been associated with extrinsic skin aging. Among many others, ultraviolet (UV) light has been studied the most extensively, while the detrimental effects of airborne pollutants such as particulate matter (PM) have recently been highlighted. Extrinsic skin aging due to UV light is also known as photoaging, and photoaging-associated hyperpigmentation disorders are very common and distressing in Asians [6,7,11]. As a result, supported by solid documentation on the molecular processes implicated in cutaneous changes induced by UV light, prevention of photoaging has long been a major subject of study in Eastern countries.

In addition to UV light, recent research has suggested a link between airborne PM exposure and skin aging [25]. It has been previously demonstrated that PM can penetrate the skin barrier and initiate a series of inflammatory reactions [24,26]. This inflammatory cascade induced by exposure to PM was found to be coupled with an increase in intracellular oxidative stress [26]. Furthermore, induction of apoptosis was demonstrated in a three-dimensional skin culture model [26], and clinically observed aggravation of cutaneous hyperpigmentation [27] has been reported.

To date, various substances, including natural compounds, have been identified to be efficacious in the prevention of skin aging, but the demand for additional agents able to protect the skin from extrinsic aging is continuously increasing as a combination approach would be far more effective [28,29,30,31]. UDCA, a natural, hydrophilic, nontoxic bile acid, could be a highly beneficial ingredient in skin and beauty products given that natural biomaterials are often considered appealing in terms of health and safety. In the skin, UDCA was reported to improve psoriasis, possibly by suppressing phospholipase A2 activity [16]. Recently, in mice, the beneficial effects of UDCA on age-related adiposity by reducing peroxisome proliferator-activated receptor-γ and inflammatory cytokines, such as TNF-α, IL-1, and CCL-2, were reported [17]. Most importantly, UDCA is thought to possess an antioxidant property [32]. Taken together, we hypothesized that UDCA may protect the skin cells exposed to UV light and PM by reducing the oxidative stress.

Our results indicate that UDCA prevents cellular events in human skin caused by the exposure to UV light or PM via the reduction of both intracellular oxidative stress and cutaneous inflammation. It has been thoroughly demonstrated that exposure to UV radiation produces an inflammatory response in the skin [33]. UV activates NF-κB in human skin fibroblasts and thus induces both the expression and release of proinflammatory cytokines, such as IL-1α and TNF-α, subsequently leading to the production of MMPs [34,35]. Moreover, UV irradiation is strongly associated with increased intracellular ROS production in the skin, which induces cellular senescence [1]. Cellular senescence is a crucial aging mechanism, and senescent cells exhibit paracrine activities on neighboring cells and tissues through a senescence-associated secretory phenotype (SASP), including proinflammatory factors [36,37]. Also, exposure to PM is known to increase ROS in the skin and elicit an inflammatory reaction in a similar fashion to UV light [24,26]. In the present study, we have demonstrated that UDCA attenuates UV- and PM-induced increases in both intracellular ROS and SASP factors. In particular, downregulation of IL-1α, TNF-α, IL-8, and AP-1 complex (c-Fos and c-Jun), which is a well-known transcription factor targeting the MMP-1 promoter region, was observed [37]. UDCA could also effectively downregulate SASPs such as COX2, MMP-1, and MMP-3, which were induced by UVA irradiation, while it restored procollagen I, which was reduced by UVA. Moreover, the expression of MAP kinases induced by UV was downmodulated by UDCA. These findings indicate that UDCA can mitigate both oxidative stress and cutaneous inflammation, which are the two main mechanisms of extrinsic aging.

We also showed that UDCA decreases melanin content in normal human melanocytes (NHMs). It has been previously reported that three-dimensional cocultures of melanocytes with photoaged human dermal fibroblasts (HDFs) results in increased melanogenesis [12]. In fact, there exists strong evidence that fibroblasts play critical roles in the development or modulation of skin hyperpigmentation disorders [13,38,39,40,41,42,43], and that UVB-irradiated HDFs directly promote melanin synthesis in melanocytes [13,41]. Thus, we tested whether UDCA inhibits melanogenesis in NHMs cocultured with UVB-irradiated HDFs. As expected, UDCA decreased the melanin content more profoundly in the coculture condition compared to the monoculture of NHMs, suggesting that UDCA could mitigate extrinsic aging-associated disordered hyperpigmentation to a higher extent. These findings are also supported by the observation that UV-induced release of paracrine melanogenic cytokines by dermal fibroblasts and keratinocytes was attenuated by UDCA treatment. Taken together, UDCA could inhibit both intrinsic melanin synthesis in melanocytes and environmentally stimulated melanogenesis, in which fibroblasts and keratinocytes act as mediators of cell signaling.

Although we have demonstrated that UDCA prevents hyperpigmentation associated with extrinsic aging, there inevitably are certain obstacles to overcome before it can actually be used for dermatologic purposes. First, the route of UDCA administration should be established. It has to be determined whether UDCA should be consumed as a functional food or developed as a topical formulation or cosmetic ingredient. Because it is still unknown how much UDCA should be taken orally so that the UDCA concentration can reach a therapeutic dose in the skin, and to minimize the potential for systemic side effects, topical application of UDCA would be preferred. Accordingly, a topical formulation of UDCA is currently under development for use in the market. Nevertheless, future studies are warranted to confirm the clinical efficacy of UDCA in the prevention of extrinsic skin aging and hyperpigmentation, and to find the right dosage in order to achieve the desired effects.

## 5. Conclusions

UDCA can effectively attenuate increased intracellular oxidative stress and melanin synthesis by exposure to UV light and PM. Given the intractable clinical course of cutaneous hyperpigmentation caused by environmental factors in Asians, both topical and systemic administration of UDCA could potentially be a safe therapeutic dosing approach for this agent, which could also have other health benefits due to its antioxidant properties.

## Figures and Tables

**Table 1:** List of primers used for quantitative Real-Time Polymerase Chain Reaction (qRT-PCR)

Name	Accession Number	Forward (5′ to 3′)	Reverse (5′ to 3′)
IL-1α	NM_000575.5	AGGGCGTCATTCAGGATGAA	CGCCAATGACTCAGAGGAAGA
IL-8	NM_000584.4	AACCCTCTGCACCCAGTTTTC	ACTGAGAGTGATTGAGAGTGGAC
TNF-α	NM_000594.4	AGCTGCCCCTCAGCTTGAG	CCCAGGGACCTCTCTCTAATCA
RPLP0	NM_001002.4	GGCGACCTGGAAGTCCAACT	CCATCAGCACCACAGCCTTC
MITF	NM_000248.4	TCTACCGTCTCTCACTGGATTGG	GCTTTACCTGCTGCCGTTGG
Tyrosinase	NM_000372.5	GGCCTCAATTTCCCTTCACA	CAGAGCACTGGCAGGTCCTAT
MMP-1	NM_001145938.2	CTCTGGAGTAATGTCACACCTCT	TGTTGGTCCACCTTTCATCTTC
MMP-3	NM_002422.5	CGGTTCCGCCTGTCTCAAG	CGCCAAAAGTGCCTGTCTT

## Data Availability

All data is contained within the article.

## References

[1] Burke K.E. (2010). Photoaging: The role of oxidative stress. G. Ital. Derm. Venereol.

[2] Gilchrest B.A. (1989). Skin aging and photoaging: An overview. J. Am. Acad. Derm..

[3] Kammeyer A., Luiten R.M. (2015). Oxidation events and skin aging. Ageing Res. Rev..

[4] Fisher G.J., Kang S., Varani J., Bata-Csorgo Z., Wan Y., Datta S., Voorhees J.J. (2002). Mechanisms of photoaging and chronological skin aging. Arch. Derm..

[5] Rabe J.H., Mamelak A.J., McElgunn P.J., Morison W.L., Sauder D.N. (2006). Photoaging: Mechanisms and repair. J. Am. Acad. Derm..

[6] Chung J.H. (2003). Photoaging in Asians. Photodermatol. Photoimmunol. Photomed..

[7] Won K.H., Lee S.H., Lee M.H., Rhee D.Y., Yeo U.C., Chang S.E. (2016). A prospective, split-face, double-blinded, randomized study of the efficacy and safety of a fractional 1064-nm Q-switched Nd:YAG laser for photoaging-associated mottled pigmentation in Asian skin. J. Cosmet. Laser Ther..

[8] Hüls A., Sugiri D., Fuks K., Krutmann J., Schikowski T. (2019). Lentigine Formation in Caucasian Women-Interaction between Particulate Matter and Solar UVR. J. Investig. Derm..

[9] Dijkhoff I.M., Drasler B., Karakocak B.B., Petri-Fink A., Valacchi G., Eeman M., Rothen-Rutishauser B. (2020). Impact of airborne particulate matter on skin: A systematic review from epidemiology to in vitro studies. Part. Fibre Toxicol.

[10] Ryu Y.S., Kang K.A., Piao M.J., Ahn M.J., Yi J.M., Bossis G., Hyun Y.M., Park C.O., Hyun J.W. (2019). Particulate matter-induced senescence of skin keratinocytes involves oxidative stress-dependent epigenetic modifications. Exp. Mol. Med..

[11] Ho S.G., Chan H.H. (2009). The Asian dermatologic patient: Review of common pigmentary disorders and cutaneous diseases. Am. J. Clin. Derm..

[12] Duval C., Cohen C., Chagnoleau C., Flouret V., Bourreau E., Bernerd F. (2014). Key regulatory role of dermal fibroblasts in pigmentation as demonstrated using a reconstructed skin model: Impact of photo-aging. PLoS ONE.

[13] Wang Y., Viennet C., Robin S., Berthon J.Y., He L., Humbert P. (2017). Precise role of dermal fibroblasts on melanocyte pigmentation. J. Derm. Sci..

[14] Lapenna D., Ciofani G., Festi D., Neri M., Pierdomenico S.D., Giamberardino M.A., Cuccurullo F. (2002). Antioxidant properties of ursodeoxycholic acid. Biochem Pharm..

[15] Rodrigues C.M., Steer C.J. (2001). The therapeutic effects of ursodeoxycholic acid as an anti-apoptotic agent. Expert Opin. Investig. Drugs.

[16] Itoh S., Kono M., Akimoto T. (2007). Psoriasis treated with ursodeoxycholic acid: Three case reports. Clin. Exp. Derm..

[17] Oh A.R., Bae J.S., Lee J., Shin E., Oh B.C., Park S.C., Cha J.Y. (2016). Ursodeoxycholic acid decreases age-related adiposity and inflammation in mice. BMB Rep..

[18] Roma M.G., Toledo F.D., Boaglio A.C., Basiglio C.L., Crocenzi F.A., Sanchez Pozzi E.J. (2011). Ursodeoxycholic acid in cholestasis: Linking action mechanisms to therapeutic applications. Clin. Sci..

[19] Moon H.R., Jung J.M., Kim S.Y., Song Y., Chang S.E. (2020). TGF-β3 suppresses melanogenesis in human melanocytes cocultured with UV-irradiated neighboring cells and human skin. J. Derm. Sci..

[20] Debacq-Chainiaux F., Borlon C., Pascal T., Royer V., Eliaers F., Ninane N., Carrard G., Friguet B., de Longueville F., Boffe S. (2005). Repeated exposure of human skin fibroblasts to UVB at subcytotoxic level triggers premature senescence through the TGF-beta1 signaling pathway. J. Cell Sci..

[21] Dobrzynska I., Szachowicz-Petelska B., Skrzydlewska E., Figaszewski Z.A. (2016). Effects of UVB Radiation on the Physicochemical Properties of Fibroblasts and Keratinocytes. J. Membr. Biol..

[22] Lei L., Zeng Q., Lu J., Ding S., Xia F., Kang J., Tan L., Gao L., Kang L., Cao K. (2017). MALAT1 participates in ultraviolet B-induced photo-aging via regulation of the ERK/MAPK signaling pathway. Mol. Med. Rep..

[23] Yang Y., Li S. (2015). Dandelion Extracts Protect Human Skin Fibroblasts from UVB Damage and Cellular Senescence. Oxid. Med. Cell Longev..

[24] Jin S.P., Li Z., Choi E.K., Lee S., Kim Y.K., Seo E.Y., Chung J.H., Cho S. (2018). Urban particulate matter in air pollution penetrates into the barrier-disrupted skin and produces ROS-dependent cutaneous inflammatory response in vivo. J. Derm. Sci..

[25] McDaniel D., Farris P., Valacchi G. (2018). Atmospheric skin aging-Contributors and inhibitors. J. Cosmet. Derm..

[26] Magnani N.D., Muresan X.M., Belmonte G., Cervellati F., Sticozzi C., Pecorelli A., Miracco C., Marchini T., Evelson P., Valacchi G. (2015). Skin Damage Mechanisms Related to Airborne Particulate Matter Exposure. Toxicol. Sci..

[27] Nakamura M., Morita A., Seité S., Haarmann-Stemmann T., Grether-Beck S., Krutmann J. (2015). Environment-induced lentigines: Formation of solar lentigines beyond ultraviolet radiation. Exp. Derm..

[28] Cavinato M., Waltenberger B., Baraldo G., Grade C.V.C., Stuppner H., Jansen-Dürr P. (2017). Plant extracts and natural compounds used against UVB-induced photoaging. Biogerontology.

[29] Nobile V., Michelotti A., Cestone E., Caturla N., Castillo J., Benavente-García O., Pérez-Sánchez A., Micol V. (2016). Skin photoprotective and antiageing effects of a combination of rosemary (Rosmarinus officinalis) and grapefruit (Citrus paradisi) polyphenols. Food Nutr. Res..

[30] Karapetsas A., Voulgaridou G.P., Iliadi D., Tsochantaridis I., Michail P., Kynigopoulos S., Lambropoulou M., Stavropoulou M.I., Stathopoulou K., Karabournioti S. (2020). Honey Extracts Exhibit Cytoprotective Properties against UVB-Induced Photodamage in Human Experimental Skin Models. Antioxidants.

[31] Jo K., Bae G.Y., Cho K., Park S.S., Suh H.J., Hong K.B. (2020). An Anthocyanin-Enriched Extract from Vaccinium uliginosum Improves Signs of Skin Aging in UVB-Induced Photodamage. Antioxidants.

[32] Chen Y.S., Liu H.M., Lee T.Y. (2019). Ursodeoxycholic Acid Regulates Hepatic Energy Homeostasis and White Adipose Tissue Macrophages Polarization in Leptin-Deficiency Obese Mice. Cells.

[33] D’Orazio J., Jarrett S., Amaro-Ortiz A., Scott T. (2013). UV radiation and the skin. Int. J. Mol. Sci..

[34] Fisher G.J., Datta S.C., Talwar H.S., Wang Z.Q., Varani J., Kang S., Voorhees J.J. (1996). Molecular basis of sun-induced premature skin ageing and retinoid antagonism. Nature.

[35] Kohl E., Steinbauer J., Landthaler M., Szeimies R.M. (2011). Skin ageing. J. Eur. Acad. Derm. Venereol..

[36] Coppe J.P., Desprez P.Y., Krtolica A., Campisi J. (2010). The senescence-associated secretory phenotype: The dark side of tumor suppression. Annu. Rev. Pathol..

[37] Toutfaire M., Bauwens E., Debacq-Chainiaux F. (2017). The impact of cellular senescence in skin ageing: A notion of mosaic and therapeutic strategies. Biochem. Pharm..

[38] Byun J.W., Park I.S., Choi G.S., Shin J. (2016). Role of fibroblast-derived factors in the pathogenesis of melasma. Clin. Exp. Derm..

[39] Hirobe T., Hasegawa K., Furuya R., Fujiwara R., Sato K. (2013). Effects of fibroblast-derived factors on the proliferation and differentiation of human melanocytes in culture. J. Derm. Sci..

[40] Kovacs D., Cardinali G., Aspite N., Cota C., Luzi F., Bellei B., Briganti S., Amantea A., Torrisi M.R., Picardo M. (2010). Role of fibroblast-derived growth factors in regulating hyperpigmentation of solar lentigo. Br. J. Derm..

[41] Salducci M., Andre N., Guere C., Martin M., Fitoussi R., Vie K., Cario-Andre M. (2014). Factors secreted by irradiated aged fibroblasts induce solar lentigo in pigmented reconstructed epidermis. Pigment Cell Melanoma Res..

[42] Yamaguchi Y., Itami S., Watabe H., Yasumoto K., Abdel-Malek Z.A., Kubo T., Rouzaud F., Tanemura A., Yoshikawa K., Hearing V.J. (2004). Mesenchymal-epithelial interactions in the skin: Increased expression of dickkopf1 by palmoplantar fibroblasts inhibits melanocyte growth and differentiation. J. Cell Biol..

[43] Yoon J.E., Kim Y., Kwon S., Kim M., Kim Y.H., Kim J.H., Park T.J., Kang H.Y. (2018). Senescent fibroblasts drive ageing pigmentation: A potential therapeutic target for senile lentigo. Theranostics.

